# Fermented *Astragalus* and its metabolites regulate inflammatory status and gut microbiota to repair intestinal barrier damage in dextran sulfate sodium-induced ulcerative colitis

**DOI:** 10.3389/fnut.2022.1035912

**Published:** 2022-11-14

**Authors:** Junxiang Li, Yingchun Ma, Xiaofeng Li, Yafei Wang, Zeqi Huo, Yang Lin, Jiaru Li, Hui Yang, Zhiming Zhang, Pingrong Yang, Chunjiang Zhang

**Affiliations:** ^1^School of Pharmacy, Lanzhou University, Lanzhou, China; ^2^Gansu Institute for Drug Control, Lanzhou, China; ^3^School of Life Sciences, Lanzhou University, Lanzhou, China; ^4^Key Laboratory of Cell Activities and Stress Adaptations, Ministry of Education, Lanzhou University, Lanzhou, China; ^5^Gansu Key Laboratory of Biomonitoring and Bioremediation for Environmental Pollution, Lanzhou University, Lanzhou, China; ^6^Gansu Provincial Hospital of Traditional Chinese Medicine (TCM), Lanzhou, China

**Keywords:** *Astragalus*, fermentation, inflammation, intestinal barrier, gut microbiota

## Abstract

Fermentation represents an efficient biotechnological approach to increase the nutritional and functional potential of traditional Chinese medicine. In this study, *Lactobacillus plantarum* was used to ferment traditional Chinese medicine *Astragalus*, the differential metabolites in the fermented *Astragalus* (FA) were identified by ultra-performance liquid chromatography-Q Exactive hybrid quadrupole-Orbitrap mass spectrometry (UPLC-Q-Exactive-MS), and the ameliorating effect of FA on dextran sulfate sodium (DSS)-induced colitis in mice were further explored. The results showed that 11 differential metabolites such as raffinose, progesterone and uridine were identified in FA, which may help improve the ability of FA to alleviate colitis. Prophylactic FA supplementation effectively improved DAI score, colon length and histopathological lesion in DSS-treated mice. The abnormal activation of the intestinal immune barrier in mice was controlled after FA supplementation, the contents of myeloperoxidase (MPO) and IgE were reduced and the contents of IgA were increased. The intestinal pro-inflammatory factors TNF-α, IL-1β, IL-6, and IL-17 were down-regulated and the anti-inflammatory factors IL-10 and TGF-β were up-regulated, suggesting that FA can intervene in inflammatory status by regulating the balance of Th1/Th2/Th17/Treg related cytokines. In addition, FA supplementation modified the structure of the intestinal microbiota and enriched the abundance of *Akkermansia* and *Alistipes*, which were positively associated with the production of short-chain fatty acids. These microbes and their metabolites induced by FA also be involved in maintaining the intestinal mucosal barrier integrity by affecting mucosal immunity. We observed that intestinal tight junction protein and mucous secreting protein ZO-1, occludin, and MUC2 genes expression were more pronounced in mice supplemented with FA compared to unfermented *Astragalus*, along with modulation of intestinal epithelial cells (IECs) apoptosis, verifying the intestinal mucosal barrier repaired by FA. This study is the first to suggest that FA as a potential modulator can more effectively regulate the inflammatory status and gut microbiota to repair the intestinal barrier damage caused by colitis.

## Introduction

Ulcerative colitis (UC) is chronic and intermittent inflammatory bowel disease of unknown etiology. Abdominal pain, diarrhea, bloody stools and weight loss are the main manifestations. UC has long been recognized in western countries. Recently, the incidence of UC is continually increasing in Asia including China, which is considered to be a result of the westernization of their lifestyle and diet (1–3). At present, UC is a complex and multifactorial disease caused by the interaction of genetics, environmental factors and host immune factors (4). There is no cure for UC, and current drugs usually have the potential to cause a range of adverse effects such as drowsiness, gastrointestinal complications and renal disturbances (5). Therefore, an alternative for effective, safe and long-term therapy or prevention of UC may be necessary.

The pathogenesis of UC, which has not been fully elucidated, involves the response of the activation of the mucosal immune system, barrier disruption and the change in gut microbiota (6). However, the key to the eventual development of UC and its clinically devastating effects such as inflammation and tissue damage is the disruption of intestinal barrier. The initial activation of the intestinal mucosal immune response is the direct cause of the onset and progression process. Therein the Th1/Th2/Th17/Treg related cytokines imbalance is considered as a prime pathway of inflammatory responses in intestinal mucosa during UC. Cytokines imbalance changes the circulating level of immunoglobulins such as immunoglobulin E (IgE) and immunoglobulin A (IgA) in the body, which marks the abnormal state of the immune system. In addition, in-depth studies on the relationship between immunity and gut microbiota have revealed the important impact of microbiota imbalance on intestinal mucosal integrity in UC (7, 8). The differences in gut microbiota composition between UC patients and healthy individuals have been documented (4). An increase in phylum *Proteobacteria* is the most consistent result of UC (9, 10). Along with an increase in the abundance of species from the phylum *Proteobacteria*, the abundance of the phyla *Firmicutes* and *Bacteroidetes* was decreased (8). Moreover, previous evidences have supported that short chain fatty acids (SCFAs), as key molecules between the microbes and the host immune system, can be produced by the gut microbiota (11, 12). Thus, bacteria capable of producing SCFAs have attracted extensive attention. Overall, UC leads to a proinflammatory shift in the gut microenvironment that disrupts the immune and biological barrier function of the intestinal mucosa (13). This process can exacerbate susceptibility in the gut. Numerous studies have observed that the tight junctions and adherence junctions of intestinal epithelial cells (IECs) are absent in UC models, which promotes the invasion of leaky intestinal epithelium by antigenic foreign bodies such as pathogenic bacteria, and further induces apoptosis of IECs (14–16).

The main pathogenesis of UC has been highlighted. Aiming at these mechanisms to solve the problem of UC is the research hotspot. Currently, numerous studies have demonstrated the role of dietary ingredients on UC prevention and therapy is perhaps the most noteworthy. For example, plenty of plant-based diets which are rich in bioactive compounds such as polysaccharides, polyphenols, alkaloids and terpenes are beneficial for UC because they have ideal anti-inflammatory, immunomodulatory properties or help regulate intestinal microbiota (17–23). In general, the mechanisms of the protective effect of plant-based diets are based on the reduction of oxidative stress, cytokine secretion, and regulation of gut microbiota in response to intestinal barrier dysfunction.

In recent years, the application of traditional Chinese medicines (TCMs) by submerged fermentation of probiotics has become a hot topic that widens the boundaries of TCMs. Microbes and their metabolic products can regulate the bioactive products of TCMs, there is a close relationship between microorganisms and TCMs consequently (24). Previous studies have shown that fermentation of TCMs mediated by microorganisms can synthesize important microbial and vegetative secondary metabolites which degrade macromolecular organic substances into small active compounds and increase the therapeutic effect (25–27). Moreover, fermentation can also reduce the adverse effects and toxicity of TCMs containing cytotoxic compounds such as heavy metals, toxic glycosides and toxic proteins (24, 26, 28). *Astragalus*, a well-known edible and medicinal plant widely distributed in China, has attracted multitudes of research attention due to its effective impact on inflammatory and immune response related diseases such as influenza, fever, sore throat, rheumatism and pneumonia (29–32). Noteworthy, Xuan Fei Hua Zhuo mixture, which is mainly composed of *Astragalus* has been recommended for the prevention of COVID-19 by Gansu Province Food and Drug Administration. *Astragalus* contains relatively high quantities of flavonoids, polysaccharides, organic acids, saponins and some trace elements, which exhibit anti-inflammatory (33), antioxidant (34), antiviral (31), anticancer (35) and improve the immunity of organism (36). Nevertheless, the recalcitrance of plant cell wall and the absorption of macromolecular substances are still obstacles to the development of the clinical potential of *Astragalus* (24), and fermentation would appear to be a novel strategy for the improvement of *Astragalus* utilization efficiency. Furthermore, fermentation may change the properties of the *Astragalus*. As recently evidenced, utilizing *Lactobacillus plantarum* (*L. Plantarum*) to ferment the *Astragalus* improves broiler growth performance, increases serum antioxidant status and reduces fecal harmful microbes of broilers (37). However, it is not clear whether *Astragalus* after fermentation can more effectively protect the intestinal barrier damage in UC by regulating inflammation, immunity and gut microbiota changes.

In the present work, we used *L. plantarum* LZU-J-TSL6, LZU-S-ZCJ isolated from fermented food JiangShui and yak yogurt in northwest China to ferment *Astragalus* and obtained the fermented *Astragalus* (FA). In order to clarify the microbial and vegetative secondary metabolites produced by fermentation, we analyzed the metabolic profiles of aqueous extract of *Astragalus* (A) and FA by ultra-performance liquid chromatography-Q Exactive hybrid quadrupole-Orbitrap mass spectrometry (UPLC-Q-Exactive-MS). Moreover, we initially evaluated the potential of FA in preventing the progression of UC, using dextran sulfate sodium (DSS) induced colitis in the murine model. Further, we studied the reversal effect of FA on the intestinal inflammatory status and gut microbiota imbalance in UC mice *via* detecting immunological markers, cytokines and gut microbiota. Finally, we examined the tight junction proteins, mucous secreting protein, and IECs apoptosis to demonstrate that FA restores the integrity of the intestinal mucosal barrier by modulating mucosal immune and biological barrier. This study would provide basic evidence to understand the potential value and mechanism of FA, and bring a new perspective to the prevention and therapy of UC.

## Materials and methods

### Preparation of *Astragalus* and fermented *Astragalus*

*L. plantarum* LZU-J-TSL6 and LZU-S-ZCJ were isolated from JiangShui and yogurt, respectively. The strains were identified by 16S rRNA gene sequence analysis (Supplementary Tables 1, 2) and were deposited in the Guangdong Microbial Culture Collection Center (GDMCC), Guangzhou, China, under the accession number GDMCC 61242 and GDMCC 61402. *Astragalus* was purchased by Deshengtang Group Co., LTD., (Lanzhou, China). The following are the preparation details:

**A:** An appropriate amount of *Astragalus* powder was extracted two times with 10 volumes of distilled water and retained the filtrate. Concentrated the filtrate to a specific concentration (0.5 g/mL) and the supernatant was collected by centrifugation and sterilized.

**FA:** The final concentration of *L. plantarum* LZU-J-TSL6, LZU-S-ZCJ in the culture was approximately 10^10^ CFU/mL. Next, *L. plantarum* LZU-J-TSL6, LZU-S-ZCJ were mixed at a ratio of 3:1 and added into A with 3% initial inoculum. After that, fermented at 36°C for 36 h. Next, the supernatant was sterilized and centrifuged to obtain FA. It is worth emphasizing that FA does not contain living microorganisms.

### Detection of the differential metabolites in fermented *Astragalus* with ultra-performance liquid chromatography-Q exactive hybrid quadrupole-Orbitrap mass spectrometry

Samples and 500 μL methanol solution were transferred into 2 mL centrifuge tubes. The mixtures were centrifuged at 12,000 rpm at 4°C for 10 min. Transferred, concentrated and dried the supernatant. 150 μL of 4 ppm of 2-Chloro-L-phenylalanine solution (as an internal standard) was added to redissolve the samples. The supernatants were filtered by 0.22 μm membrane and taken for assay.

The LC analysis was performed on a Vanquish UHPLC system (Thermo Fisher Scientific, USA) with an ACQUITY UPLC^®^ HSS T3 column (2.1 × 150 mm, 1.8 μm, Waters, Milford, MA, USA). The column maintained at 40 °C. Elution was carried out with a mobile phase of 5 mM acetonitrile (A) and ammonium (B) for negative model and 0.1% formic acid in acetonitrile (C) and 0.1% formic acid in water (D) for positive model at a flow rate of 0.25 mL/min. The injection volume was 2 μL of each sample. Separation was conducted under the following gradient: 0–1 min, 2% A/C; 1–9 min, 2–50% A/C; 9–12 min, 50–98% A/C; 12–13.5 min, 98% A/C; 13.5–14 min, 98–2% A/C; 14–20 min, 2% C (14–17 min, 2% A).

The MS/MS detection of metabolites was performed on Q Exactive Focus (Thermo Fisher Scientific, USA) with an electrospray ionization (ESI) interface. The parameters were as follows: sheath gas pressure of 30 Arb, aux gas flow of 10 Arb, capillary temperature, 325 °C, spray voltage of 3.50 kV (positive) and --2.50 kV (negative), respectively. The Orbitrap analyzer scanned over a mass range of m/z 81--1,000 for full scan at a resolution of 70,000. Data dependent acquisition (DDA) MS/MS experiments were performed with higher energy collisional dissociation (HCD) scan. The normalized collision energy was 30 eV. Dynamic exclusion was implemented to remove some unnecessary information in MS/MS spectra. The resulting raw data were converted into mzXML format using ProteoWizard (version 3.0)^1^ and processed with an internal program, which was developed using R and based on XCMS for peak detection, extraction, alignment and integration. Then, metabolites were annotated using BiotreeDB (the internal MS2 database). The cutoff value of annotation was set at 0.3.

### Animal experimental design

**FIGURE 1:**
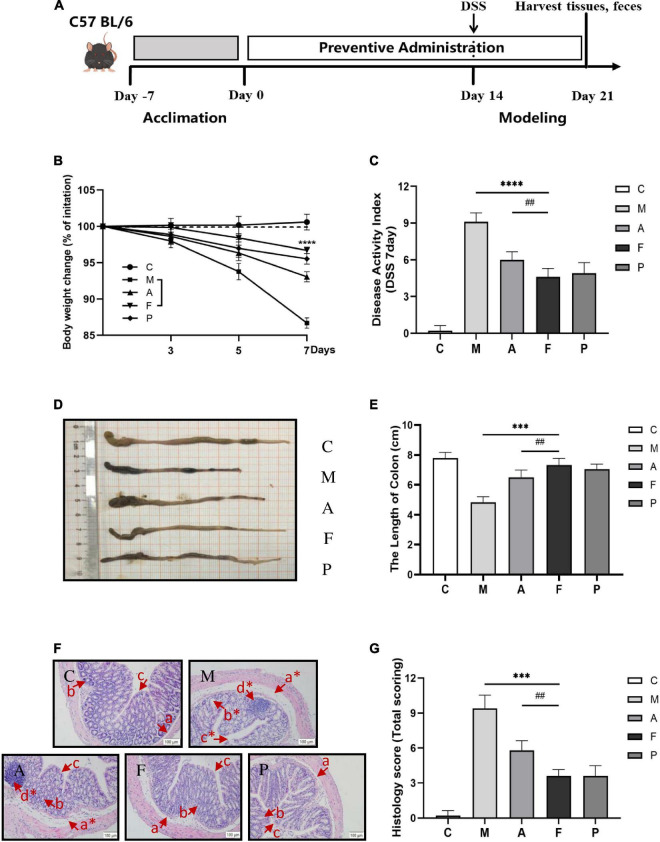
FA alleviated the severity of DSS-induced colitis in mice: **(A)** schematic diagram of the animal experiment; **(B)** FA ameliorated the body weight loss associated with the dextran sulfate sodium (DSS)-induced colitis in mice; **(C)** DAI score; **(D)** representative images of colon tissues; **(E)** colonic lengths from different groups; **(F)** representative Hematoxylin and Eosin (H&E)-stained colon sections are shown at 20 × magnification. The same locations are marked with the same symbols. (a) Edema in loose connective tissue (*moderate edema); (b) crypt and goblet cell (*crypt distortion and loss of goblet cells); (c) epithelial layer (*broken epithelial layer); (d) inflammatory infiltration; **(G)** the histological score of the colons. Data are mean ± SD (*n* = 6). ^***^P ≤ 0.001 and ^****^P ≤ 0.0001 vs. the M group; ^##^*P* ≤ 0.01 vs. the A group.

The animal experimental schematic diagram is shown in Figure 1A. Forty female C57BL/6 mice (7 weeks old) were obtained from Lanzhou veterinary research institute (Lanzhou, China) and housed in a specific pathogen-free facility with *ad libitum* access to food and water. After a 7-day acclimation, the mice were randomly divided into one of the following five experimental groups (8 mice per group): C group (control group; administration of saline prophylactically; without being exposed to DSS); M group (model group; administration of saline prophylactically); A group (aqueous extract of *Astragalus* group; administration of A prophylactically, 5 g/kg/day); F group [fermentation group; administration of F prophylactically, 5 g/kg/day); P group (positive drug group; administration of 5-aminosalicylic acid (5-ASA) prophylactically, 0.67 g/kg/day].

After 2 weeks of prophylactical supplementation, all mice (except mice from the C group) were exposed to DSS treatment (molecular weight 36–50 kDa; Yeasen Biotechnology Co., Ltd.) in their drinking water (3.5%) for seven consecutive days. The body weight, stool consistency and rectal bleeding were assessed daily and used for disease activity index (DAI) analysis (2, 19). At the end of the experiment, the blood samples were collected from eyes and centrifuged at 1,200 g for 10 min to obtain serum. After the mice were sacrificed by cervical dislocation, the length of the colon was measured from the colon-cecal junction to the anus. 2 cm of colon was fixed for histological analysis, and then the rest colons and feces were collected and stored at –80^°^C for further analysis. All animal experiments were handled following the Regulations for the Administration of Affairs Concerning Experimental Animals in China^2^ and were approved by the Ethics Committee of Lanzhou University (approval number: EAF2022041)].

### Histological analysis

Fixed colon tissues with 10% neutral formalin fixative for 48 h. The fixed tissues were dehydrated, embedded in paraffin, sectioned and stained with hematoxylin and eosin (H&E). The tissue histopathology was observed under a microscope. The histological and pathological scores were conducted according to previous studies based on the degree of inflammation, mucosal damage, crypt damage and range of pathological changes (11, 38).

### Measurement of myeloperoxidase and diamine oxidase

Colon tissues rinsed with pre-cooled PBS (0.01 M, pH = 7.4) were cut into pieces and homogenized in ice cold normal saline solution with the help of a homogenizer. The supernatant of the homogenate centrifuged at 5,000 rpm for 10 min was collected for testing. BCA protein assay was used to determine the protein content in the supernatant. The level of MPO in colon tissue was analyzed using enzyme linked immunosorbent assay (ELISA, Nanjing Jiancheng Bioengineering Institute, Nanjing, China) kits following the instructions. The MPO level was expressed in U g^–1^ wet tissue. To measure the diamine oxidase (DAO) level in serum, the serum samples were thawed at 37°C for 1 h, and DAO was assessed using an ELISA kit (Nanjing Jiancheng Bioengineering Institute, Nanjing, China) following the instructions. The samples were added into a 96-well plate and run on a microplate reader (Bio-Rad iMARKTM, Shanghai, China). Each experiment was repeated three times, and average optical density values were taken as statistics.

### Measurement of cytokines

The levels of inflammatory cytokines (TNF-α, IL-1β, IL-6, IL-10, IL-17, TGF-β) in colon tissue were analyzed using ELISA (Nanjing Jiancheng Bioengineering Institute, Nanjing, China) kits following the instructions. And immunoglobulins (IgE, IgA) in serum were analyzed using ELISA (Nanjing Jiancheng Bioengineering Institute, Nanjing, China) kits following the instructions. Each group had three complex holes, and average optical density values were taken as statistics.

### Analysis of gut microbiota

The feces of samples were sent to Suzhou PANOMIX Biomedical Tech Co., Ltd., China for 16S rRNA gene sequencing. Extracted total genome DNA from samples using CTAB method (Qiagen, Hilden, Germany). The concentration and integrity of DNA were assessed using Nanodrop (Thermo Fisher Scientific, USA) and 1.2% agarose gel electrophoresis, respectively. According to the concentration, DNA was diluted to 1 μg/mL using sterile water. PCR primers containing a barcode (515F 5′-ACTCCTACGGGAGGCAGCA-3′, 806R 5′-GGACTACHVGGGTWTCTAAT-3′) were used to amplify the bacterial 16S rRNA variable region V4. All PCR reactions were carried out with Phusion^®^ High-Fidelity PCR Master Mix (New England Biolabs, Ipswich, USA). Purified PCR products with Qiagen Gel Extraction Kit (Qiagen, Dusseldorf, Germany). An equal amount of DNA was used to prepare sequencing libraries with the Ion Plus Fragment Library Kit (Thermo Fisher Scientific, USA) according to the manufacturer’s recommendations. Quantified by using the Qubit 2.0 Fluorometer (Thermo Fisher Scientific, USA) and Agilent Bioanalyzer 2,100 system. The qualified libraries were sequenced on the MiSeq platform with the PE250 sequencing strategy. Raw data was analyzed using the QIIME2 platform. Multiplexed single end sequencing reads (> 50,000 per sample) were imported into the QIIME2 platform. Raw reads were quality filtered, assembled, and chimeric sequences were removed using data2, which generated unique amplicon sequence variants (ASVs) instead of clustering similar sequences into traditional operational taxonomic units. Subsequently, we used the SILVA reference database classifier for the classification of ASVs with a threshold of 100% sequence similarity. Determinations of alpha and beta diversities were also conducted in QIIME 2. Non-metric multidimensional scaling (NMDS) analysis plots were generated using the “ggplot2” packages of the R software. Linear discriminant analysis (LDA) effect size (LEFSE) was used to identify genera with evident differences between groups. Only bacterial taxa that reached the LDA threshold of 2.0 and had an average relative abundance greater than 0.01% were shown.

### Detection of supported that short chain fatty acids in feces with gas chromatography-mass spectrometry

The measurement of SCFAs in feces has been previously reported (39). Samples were pipetted in 2 mL centrifuge tubes, 50 μL of 15% phosphoric acid, 100 μL of 125 μg/mL of internal standard (isocaproic acid) solution were added. The mixtures were homogenized with 400 μL of ether for 1 min and centrifuged at 4°C for 10 min at 12,000 rpm. The supernatants were taken for assay.

SCFAs in feces were analyzed using a TRACE 1,310 gas chromatography coupled to an ISQ LT mass spectrometry (Thermo Fisher Scientific, Waltham, MA, USA). The capillary gas chromatography-mass spectrometry (GC-MS) column HP-Innowax (30 cm × 0.25 mm × 0.25μm) was obtained from Agilent (Agilent Technology, USA). The temperature program of 90°C was increased to 120°C at a rate of 10°C/min, then to 150°C at 5°C/min, and then to 250°C for 2 min at 25°C/min. The injection volume was 1 μL with split injection ratio of 10:1. Helium was used as carrier gas (flow rate: 1.0 mL/min). Mass spectra were operated in electron impact ionization (EI) mode at 70 eV.

### Measurement of nucleosome

The level of nucleosome in colon tissue was assessed using ELISA (Shanghai Jianglai Bio-Technology Co., Ltd., Shanghai, China) kit following the instructions. Each group had three complex holes, and average optical density values were taken as statistics.

### Gene expression analysis by quantitative real-time polymerase chain reaction

The relative expression levels of genes related to colonic mucous barrier integrity (ZO-1, occludin and MUC2) and IECs proliferation and apoptosis (Bax, Bcl-2) were determined using quantitative real-time polymerase chain reaction (qRT-PCR). Total RNA was extracted from the colon tissues following the protocol by Servicebio, Inc. (Wuhan Servicebio Technology Co., Ltd., Wuhan, China) and reverse transcribed to cDNA using a Servicebio^®^RT First Strand cDNA Synthesis Kit (Servicebio, China). The relative expression levels of the selected genes were determined using a Bio-Rad PCR machine (Bio-Rad, USA). Thermal cycling consisted of an initial cycle of 95°C for 10 min, followed by 40 amplification cycles of 95°C for 15 s and 60°C for 30 s. The GAPDH was used as an internal gene to normalize the selected genes. And the relative expression levels were calculated using the 2^–ΔΔCt^ formula. Each sample in each group had three complex holes, and average expression values were taken as statistics. The primer sequences for all the genes (GAPDH, ZO-1, occluding, MUC2, Bax and Bcl-2) were provided in Supplementary Table 3.

### Statistics

Data were presented as the mean ± standard deviation (SD). All experiments were repeated at least 3 times by independent assays. Data sets involving more than two groups were compared by one-way analysis of variance (ANOVA), followed by Duncan’s multiple range test or Student’s *t*-test using GraphPad Prism 8.0 (GraphPad Software Inc., USA). Values of *P* < 0.05 were considered to be statistically significant.

## Results

### The main differential metabolites of fermented *Astragalus*

The main differential metabolites of FA were identified using UPLC-Q-Exactive-MS. 489 metabolites in FA and A were revealed totally according to accurate mass and MS/MS data of metabolites in MS2 databases. In order to accurately find out differential metabolites influenced after fermentation, the rigorous filtering conditions were set (Value of *P* < 0.05; VIP > 1). After filtering, the alterative metabolites were compared in FA and A. In this paper, compared to A, the differential metabolites which significantly increased in FA were listed in Table 1.

**TABLE 1:** The significantly increased metabolites in FA vs. A.

Name	A (mean ± SD)	FA (mean ± SD)	Fold change	VIP
Raffinose	4478587.75 ± 30.04	109678473.68 ± 15.35	24.49	1.16
3,4-Dihydroxybenzaldehyde	3474631.57 ± 19.44	48372996.62 ± 48.04	13.92	1.14
Guanidinosuccinic acid	29887646 ± 34.80	352365916 ± 55.93	11.79	1.10
Progesterone	30625467.38 ± 12.02	328457741.95 ± 29.96	10.72	1.15
(S)-2,3,4,5-tetrahydropyridine-2-carboxylate	73837662.41 ± 72.11	429113914.54 ± 27.71	5.81	1.07
5-Acetamidovalerate	10639177.52 ± 7.01	59933375.41 ± 25.38	5.63	1.16
(R)-5,6-Dihydrothymine	140609474.92 ± 28.85	760210903.56 ± 4.51	5.41	1.14
D-Octopine	27194635.91 ± 8.06	145209178.48 ± 13.67	5.34	1.17
Dodecanoic acid	12697478.43 ± 4.02	55378056.54 ± 14.83	4.36	1.16
Aminocaproic acid	503480071.34 ± 36.37	1795098042.55 ± 47.21	3.57	1.00
Uridine	3308065876.69 ± 36.46	7508092928.28 ± 25.12	2.27	1.04

As shown in Table 1, when comparing the effects of fermentation on the main ingredients in FA, 11 differential metabolites including Raffinose, 3,4-Dihydroxybenzaldehyde, Guanidinosuccinic acid, Progesterone, (S)-2,3,4,5-tetrahydropyridine-2-carboxylate, 5-Acetamidovalerate, (R)-5,6-Dihydrothymine, D-Octpine, Dodecanoic acid, Aminocaproic acid and Uridine were up-regulated in FA when compared to A. Based on MS2 databases, the normalized information of selected metabolites associated with these increased metabolites were listed in Supplementary Table 4. Notably, Raffinose was the only carbohydrate among these 11 metabolites, which consists of one molecule of fructose, galactose and glucose. Other carbohydrates and glycosides with these structural units decreased significantly after fermentation such as Trehalose, Melibiose, Sucrose, D-galactose and Cyanidin-3-galactoside. This may be related to biosynthesis and degradation during fermentation, which ultimately led to an increase in Raffinose. Similarly, we also found that 3,4-Dihydroxybenzaldehyde, Progesterone, Dodecanoic acid, Aminocaproic acid and Uridine were increased and a decrease in their corresponding precursor substances could be found (Supplementary Table 4). Furthermore, multiple amino acid pathways related metabolites, such as Guanidinosuccinic acid, (S)-2,3,4,5-tetrahydropyridine-2-carboxylate, 5-Acetamidovalerate, (R)-5,6-Dihydrothymine and D-Octpine were significantly produced, which were possibly involved in the metabolic process of the microorganisms in fermentation.

### Protective effect of fermented *Astragalus* on dextran sulfate sodium-induced colitis in mice

After 2 weeks of prophylactical supplementation, DSS-induced ulcerative colitis was established in C57 BL/6 mice to prove the protective effects of FA. A 25% mortality rate was observed in the M group, while no mortality could be observed in the F group. Body weight loss and shortened colon length are characteristic symptoms of ulcerative colitis. Compared with the C group, an expected decrease of 13.32% in weight was observed on day 7 of DSS administration. Pretreatment with FA appeared to ameliorate DSS-induced weight loss and the mice in the F group lost only 3.31% of their body weight. Furthermore, the effect of slowing weight loss of FA was more desirable than that of A or 5-ASA (Figure 1B). The severity of colitis progression was further assessed by DAI score. DAI score remarkably increased in the M group. Administration of FA prophylactically markedly decreased the DAI score (*P* ≤ 0.0001), which was consistent with body weight (Figure 1C). The representative images of colon tissues were shown in Figure 1D. Similarly, the severe disease progression was verified by the marked shortening of colon length in the M group (4.82 ± 0.54 cm), which was also relieved to vary degrees in the other groups. Especially, the colon length of FA treated mice was closest to that of normal mice (Figure 1E).

To further evaluate the deteriorating colonic structure changes, the histopathological conditions of colon tissue was performed. Compared to the C group, severe damage to colon could be observed in DSS-treated mice, including a marked crypt defect or necrosis, IECs abscission, inflammatory cellular infiltration (Figure 1F). Therefore, the highest histological score was calculated in the M group. FA supplementation could appreciably alleviate the pathological injury of colon by reducing inflammatory cells infiltration, restoring the epithelial structure and mucous membrane architecture. Notably, mice in the F group had significantly less colonic damage than mice in the A group (*P* ≤ 0.01), revealing that the fermentation was vital to improve the ability of A to protect mice from DSS-induced colitis (Figures 1F,G).

### Fermented *Astragalus* modulated immunity and Th1/Th2/Th17/Treg cytokines balance

**FIGURE 2:**
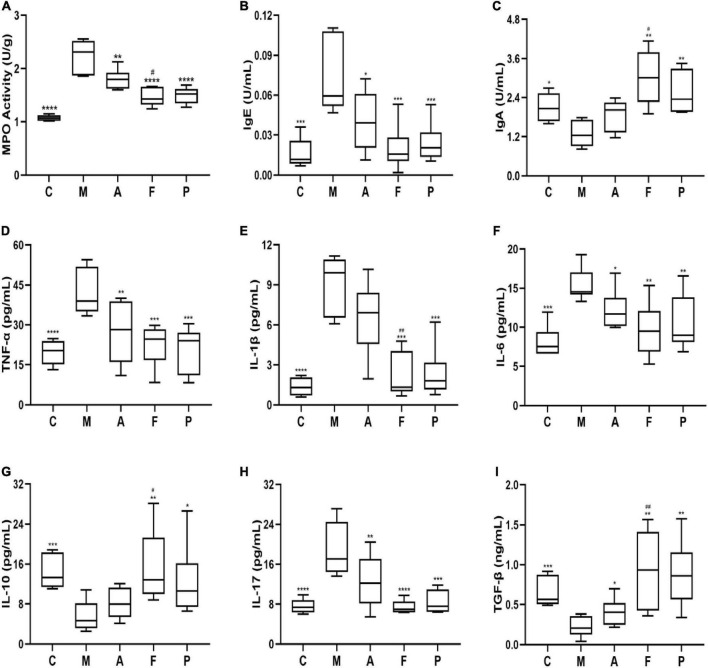
FA attenuated the colonic inflammation and modulated the inflammatory cytokines balance in DSS-induced mice: **(A)** MPO; **(B)** IgE; **(C)** IgA; **(D)** TNF-α; **(E)** IL-1β; **(F)** IL-6; **(G)** IL-10; **(H)** IL-17; **(I)** TGF-β in serum or colonic tissue. Data are mean ± SD (*n* = 5–6). *P ≤ 0.05, ^**^P ≤ 0.01, ^***^P ≤ 0.001, and ^****^P ≤ 0.0001 vs. the M group; ^#^*P* ≤ 0.05 and ^##^*P* ≤ 0.01 vs. the A group.

Preliminary analysis of colonic inflammatory response revealed a significant increase in MPO level in the M group. In contrast, samples in the other groups revealed a great reduction in MPO (Figure 2A). Simultaneously, immunoglobulins in serum of DSS-treated animals revealed a significant increase in IgE level with a decrease in IgA level suggesting inordinated humoral immune response. As previously observed, FA administration reversed the effects of DSS and appeared to maintain B lymphocyte homeostasis, as evident from IgE and IgA levels (Figures 2B,C). Notably, the effect of FA was comparable to that of positive drug (5-ASA), suggesting that FA had a strong potency to maintain the intestinal mucosal immune barrier.

To further verify the anti-inflammatory mechanisms of observed effects of FA, we detected the related inflammatory cytokines levels of Th1 (TNF-α, IL-1β, IL-6), Th2 (IL-10), Th17 (IL-17) and Treg (TGF-β) cytokines in colonic homogenate. As shown in Figures 2D–G, the levels of TNF-α, IL-1β and IL-6 in the M group were 2.14, 6.70, and 1.90 folds higher than those in normal mice, respectively. Amongst, all the prophylactical administration groups, FA significantly (*P* ≤ 0.01) inhibited the levels of TNF-α, IL-1β and IL-6 by 0.53, 0.24, and 0.63 folds compare to the M group, respectively. Interestingly, these inhibitory effects of FA were similar to 5-ASA and stronger than those of A (especially IL-1β). On the other hand, DSS decreased the levels of anti-inflammatory cytokine IL-10 in comparison with the C group, while an elevated level was observed in FA administration in comparison to the M group. Additionally, the level of IL-17 was improved by 1.49-fold in the M group in contrast to normal mice. An effective suppression for IL-17 up to 1.64-fold was observed with FA supplement, whereas A inhibited IL-17 only 1.25-fold (Figure 2H). On the contrary, the level of TGF-β in the M group was lower than that in the C group (*P* ≤ 0.001). FA supplement significantly increased the secretion of TGF-β (*P* ≤ 0.01, Figure 2I). Overall, the above results revealed that FA could more effectively modulate the balance of Th1/Th2/Th17/Treg related cytokines, which play a pivotal role in colonic inflammation and intestinal immunity.

### Fermented *Astragalus* altered the gut microbiota composition and function differently from *Astragalus* and 5-aminosalicylic acid

**FIGURE 3:**
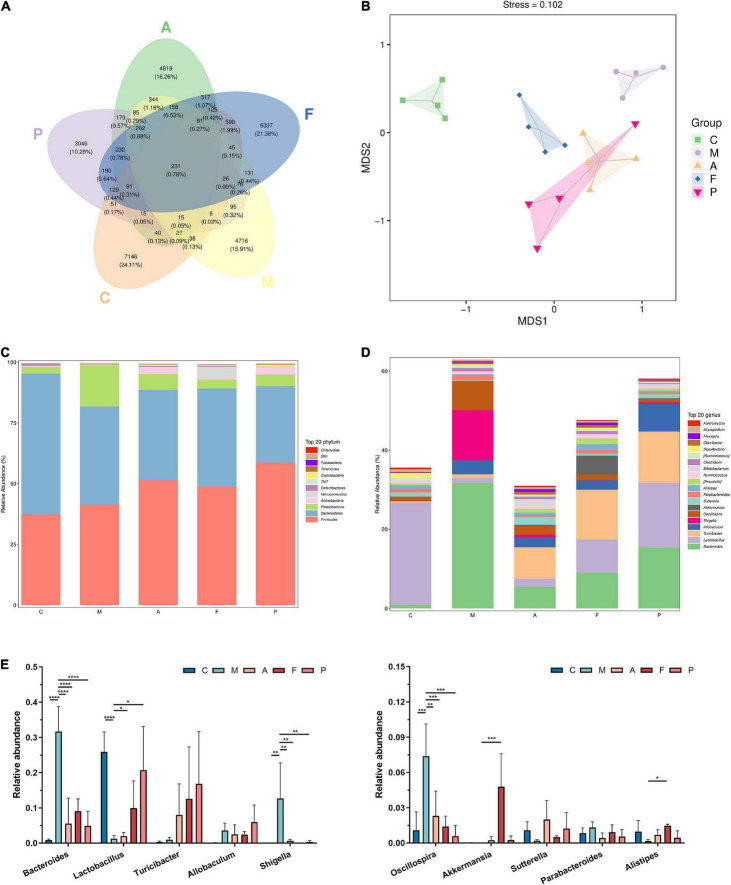
FA altered the microbial diversity and gut microbiota composition: **(A)** venn plot that illustrated the observed ASV counts in samples; **(B)** NMDS analysis based on Jaccard distance; **(C)** phylum level of the gut microbiota composition; **(D)** genus level of the gut microbiota composition; **(E)** relative abundance of the ten most predominant gut microbiota at the genus level (*n* = 4). *P ≤ 0.05, **P ≤ 0.01, ***P ≤ 0.001, and ****P ≤ 0.0001 vs. the M group.

The observed species, Chao 1index, Simpson index and Shannon index are commonly used to evaluate the richness and diversity of bacterial species. As shown in Table 2, exposure to DSS could decrease the observed species, Chao 1 index and Shannon index, while A or FA administration could increase these indices. Notably, all indices in the P group were the lowest among the five groups. Presumedly, it was related to the pharmacological action of 5-ASA and the duration of administration up to 3 weeks. It revealed that FA could reshape the microbial richness and diversity of gut bacteria, which was quite different from that of the positive drug. The mutual and unique ASVs among each group were expressed by the Venn plot in Figure 3A. 231 ASVs were mutual by all groups, with the C group having the largest count of specific ASVs (7,146). Meanwhile, 4,716, 4,819, 6,337, and 3,046 unique ASVs were observed, respectively in the M, A, F and P groups. These results agreed with the richness index and diversity index in Table 2. The β-diversity analysis was performed to assess the variance of diversity among each group. NMDS analysis based on Jaccard distance showed a segregated gut microbial structure among all groups (Figure 3B). In summary, DSS could cause structural disorder of intestinal microbiota, while FA could restore this disturbance to the greatest extent.

**TABLE 2:** The gut microbial diversity indices.

Group	Richness index		Diversity index	
	Observed species	Chao1	Simpson	Shannon
C	2865.78 ± 355.38	3099.52 ± 446.10	0.97 ± 0.01	8.09 ± 0.11
M	2033.73 ± 302.34	2304.29 ± 398.47	0.97 ± 0.02	7.43 ± 0.43
A	2244.63 ± 502.24	2546.45 ± 612.93	0.97 ± 0.01	7.71 ± 0.50
F	2817.85 ± 657.52	3076.96 ± 672.64	0.97 ± 0.02	8.12 ± 0.81
P	1315.77 ± 99.63	1459.50 ± 119.45	0.94 ± 0.02	6.32 ± 0.29

Twelve phyla were identified in fecal microbiota by 16S rRNA sequencing, and details for the groups were shown in Figure 3C. The differences mainly occurred in the three phyla with the largest proportion of gut microbiota, namely *Bacteroidetes*, *Firmicutes* and *Proteobacteria*. Compared with normal mice, the abundance of *Firmicutes* and *Proteobacteria* in the intestinal tract of DSS-treated mice increased, while the abundance of *Bacteroidetes* decreased. On the other hand, the A, FA and 5-ASA administration could decrease the abundance of *Proteobacteria* and increase the abundance of *Bacteroidetes*. In addition, increased *Actinobacteria* abundance was observed in both A and 5-ASA administration, but no change was observed in FA administration, indicating that the changes in gut microbiota caused by A, 5-ASA, FA were not consistent. It was noteworthy that the abundance of *Verrucomicrobia* in the F group was higher than that in other groups. And the high abundance of *Verrucomicrobia* appeared to be uniquely produced by FA administration.

**FIGURE 4:**
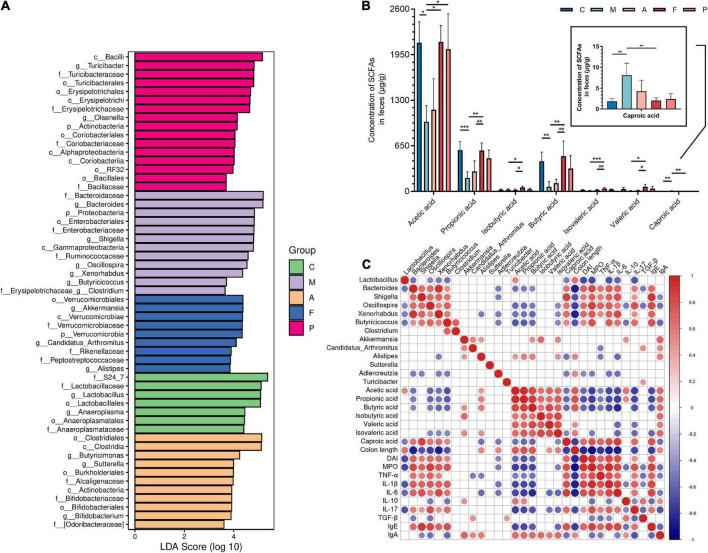
Key microbial communities characteristics and function of gut microbiota: **(A)** analysis of differences in the microbiota shown by LEfSe (linear discriminant analysis effect size); **(B)** the content of acetic acid, propionic acid, butyric acid, isobutyric acid, valeric acid, isovaleric acid and caproic acid in feces of DSS-induced mice; **(C)** correlation between key microbes, short chain fatty acids and parameters of colitis. Data are mean ± SD (*n* = 4). *P ≤ 0.05, **P ≤ 0.01, and ***P ≤ 0.001 vs. the M group; ^#^*P* ≤ 0.05 and ^##^*P* ≤ 0.01 vs. the A group. The red color denotes a positive correlation, while blue color denotes a negative correlation. The intensity of the color is proportional to the strength of Spearman correlation.

Figure 3D shows the abundance of the gut microbiota at the genus level. The main genera of the C group were concentrated in *Lactobacillus*, while the dominant genera of the M group were *Bacteroides*, *Shigella*, *Oscillospira* and *Allobaculum*. A, FA and 5-ASA administration could effectively reduce the abundance of *Bacteroides* (*P* ≤ 0.0001), *Shigella* (*P* ≤ 0.01) and *Oscillospira* (*P* ≤ 0.01), as well as increase *Lactobacillus*, *Turicibacter*, *Sutterella*, *Alistipes* (Figures 3D,E). The abundance of *Alistipes* belonging to *Bacteroidetes* appears to have increased in all three prophylactic administration groups, but this increased abundance was statistically different only in the FA group (*P* ≤ 0.05). In addition, corresponding to the changes of microbiota at the phylum level, the *Akkermansia*, which belongs to *Verrucomicrobia*, was significantly increased in the intestine of mice prevented with FA (*P* ≤ 0.001). To further investigate the influences induced by FA administration, we performed a LEfSe analysis to identify the altered bacterial taxa. We found that *Xenorhabdus*, *Butyricicoccus* and *Clostridium* were enriched by DSS treatment. Additionally, three bacterial genera including *Akkermansia* were enriched in the F group (Figure 4A).

Many intestinal microbes are associated with the production of SCFAs, and SCFAs are of great significance for the treatment of colitis and human health. Therefore, we detected the contents of seven SCFAs in feces of each group. As shown in Figure 4B, the concentration of SCFAs including acetic acid, propionic acid, butyric acid, isobutyric acid, valeric acid and isovaleric acid were reduced after DSS treatment, especially propionic acid (*P* ≤ 0.001) and butyric acid (*P* ≤ 0.01). On the contrary, the elevated content of caproic acid were observed after DSS treatment (*P* ≤ 0.01). In the F group, the content of acetic acid, propionic acid, butyric acid, isobutyric acid, valeric acid and isovaleric acid were increased, especially isovaleric acid (*P* ≤ 0.001), propionic acid (*P* ≤ 0.01) and butyric acid (*P* ≤ 0.01). Particularly, the content of these SCFAs in the F group was close to or even higher than that in the intestines of normal mice. Similarly, caproic acid content was significantly restored (*P* ≤ 0.01) after FA administration. Overall, FA has an ideal ability to regulate the biosynthesis of SCFAs, which is better than A and 5-ASA.

As previously stated, SCFAs are key molecules between gut microbiota and the host immune system. Therefore, it is necessary to probe the relationship between microbiota and SCFAs to focus on the microbes with potential functions to produce SCFAs. Further, to demonstrate the correlation between different microbes and intestinal mucosal immune function, key microbes from Figure 4A, SCFAs, and colitis parameters were explored by Spearman correlation analysis. As shown in Figure 4C. We found that the pathogenic bacteria of colitis, such as *Oscillospira* and *Xenorhabdus*, were generally negatively correlated with the production of acetic acid, propionic acid, butyric acid and isovalerate acid (*P* ≤ 0.05). The production of butyric acid and isobutyric acid was positively correlated with *Akkermansia* (*P* ≤ 0.05). *Alistipes* was positively related to the production of acetic acid, propionic acid, butyric acid and isovaleric acid (*P* ≤ 0.05). On the other hand, these microbes were associated with at least one parameter of colitis. For example, *Turicibacter* was positively correlated with TGF-β (*P* ≤ 0.05). Pathogenic bacteria of colitis such as *Shigella*, *Oscillospira* and *Xenorhabdus*, which were negatively correlated with most SCFAs, were significantly positively correlated with MPO, TNF-α, IL-1β, IL-6, IL-17, and IgE (*P* ≤ 0.05). This result might have led to a decreased colonic length and increased DAI. In contrast, *Akkermansia* had a remarkable positive correlation with IL-10 and IgA (*P* ≤ 0.05). Unlike *Akkermansia*, *Alistipes* showed a negative correlation with TNF-α and IL-6 (*P* ≤ 0.05). Therefore, it may explain the effect of FA on intestinal mucosal immune process through enrichment of *Akkermansia* and *Alistipes*.

### Fermented *Astragalus* did repair intestinal mucosal barrier structure damage

**FIGURE 5:**
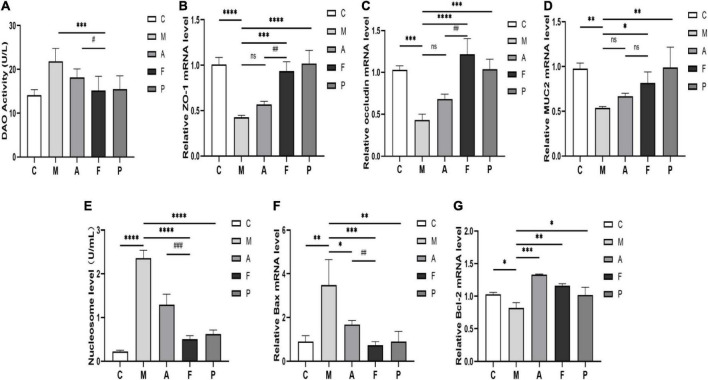
FA repaired intestinal mucosal barrier structure and ameliorated cells apoptosis in DSS-induced mice: **(A)** DAO activity in serum; **(B)** the expression of ZO-1 in colon tissue; **(C)** occludin; **(D)** MUC2; **(E)** the level of the nucleosome in colon tissue; **(F)** the expression of Bax in colon tissue; **(G)** Bcl-2. Data are mean ± SD (*n* = 3–4). *P ≤ 0.05, ^**^P ≤ 0.01, ^***^P ≤ 0.001, and ^****^P ≤ 0.0001 vs. the M group; ^#^*P* ≤ 0.05, ^##^*P* ≤ 0.01, and ^###^*P* ≤ 0.001 vs. the A group. ns, no significance.

As previously described, FA could effectively regulate the inflammatory status and the immune-related gut microbiota. Therefore, mechanical and chemical barriers of the intestinal mucosa need to be tested to verify the restorative effects of FA. Firstly, we examined the DAO activity in serum to affirm the permeability of intestinal mucosal barrier. In the M group, a significant increase in DAO activity was observed. Conversely, FA administration could change this situation effectively (*P* ≤ 0.001), which indicated the intestinal barrier integrity was better after FA prevention (Figure 5A). Then, to further identify the function of FA in protecting the damaged intestinal epithelial barrier in DSS-treated mice, the gene expression of tight junction proteins (ZO-1, occludin) and mucous secreting proteins (MUC2) were analyzed. DSS led to decreased expression of ZO-1, occludin and MUC2 genes by 0.58, 0.58, and 0.45 fold, respectively, in comparison to normal mice. On the other hand, FA prevention enhanced the expression up to 1.20, 1.82, and 0.52 fold, respectively, in comparison to the M group. It is worth noting that compared with M group, the increase of ZO-1, occludin and MUC2 genes caused by A administration was not statistically significant (Figures 5B–D). These results verified the potential of FA to promote the formation of tight junction proteins and mucus secreting proteins in IECs.

Next, we further confirmed that FA did repair the intestinal mucosal barrier by detecting the IECs apoptosis. The nucleosome levels in colon tissues were shown in Figure 5E. And the highest nucleosome level appeared in the M group, which meant that IECs underwent severe apoptosis and DNA breakage. Compared to A and 5-ASA, FA administration reversed the degree of IECs apoptosis to the greatest extent. We also examined the pro-apoptotic gene Bax and the anti-apoptotic gene Bcl-2 (Figures 5F,G). DSS significantly increased the Bax expression and decreased the Bcl-2 expression. However, FA significantly decreased Bax expression (*P* ≤ 0.001) and enhanced Bcl-2 expression in IECs (*P* ≤ 0.01). Interestingly, although not statistically different, the ameliorating effect of A on Bcl-2 appeared to be stronger than that of FA (Figure 5G). This discrepancy arised probably due to the previously mentioned functional inconsistencies in the immune and gut microbiota between A and FA. Overall, FA did eventually repair intestinal mucosal structure damage, as reflected by the desirable effect on tight junction proteins, mucins, and IECs abnormal apoptosis.

## Discussion

In this study, we used microbial fermentation technology to ferment *Astragalus*, to further produce beneficial microbial and vegetative secondary metabolites, so as to expect the FA to have a more effective effect on alleviating UC. *L. plantarum* is capable of using polysaccharides and other substances in TCMs as carbon and energy sources by producing abundant enzyme systems. These enzyme systems can help to dissolve the effective ingredients in TCMs (40). Meanwhile, the fermentation of TCMs under the action of microbial metabolism can transform the large molecule substances into small molecule metabolites. The body is easier to absorb these small metabolic compounds, which further improve or even transform the efficacy of TCMs. On the other hand, secondary metabolites produced by *L. plantarum* have a synergistic effect with the active compounds in TCMs (24, 28). For this reason, it is necessary to investigate the metabolic compounds produced in FA. Our current study found that 11 differential metabolites were up-regulated in FA. The production of these metabolites was closely related to the enzyme systems in the fermentation system, which were involved in the decomposition process of the upstream compounds and the synthesis process of the downstream compounds. Despite the availability of these metabolites, some compounds have not been extensively studied, the relationships between UC and several compounds have been established. For instance, progesterone, aminocaproic acid and uridine have been proved to contribute to the improvement of experimental colitis (41–44). Among them, a recent study has shown that uridine is a potent cell regeneration promoting factor, which means it can dominate the regeneration of IECs (45). Another case is the compound raffinose, which has been recognized as a prebiotic with positive effects on gut health, although there is no direct evidence for its relationship with UC. The above analysis of metabolites in FA could also help us partially explain why FA can effectively improve UC.

Despite the precise pathogenesis of UC is not fully understood, the mucosal necrosis, increased permeability and degenerated function of the intestinal barrier caused by inflammatory status are the key links in its pathogenesis. At present, it is considered that regulating inflammatory status and promoting intestinal barrier repair are effective methods to improve UC (46, 47). We used a DSS-induced colitis model in mice to investigate the efficacy and the underlying mechanisms of FA against UC. The present study demonstrated that FA prevention effectively attenuated some symptoms resembling clinical human colitis, including decreased body weight, reduced colon length and colonic tissue injury. Also, we set up unfermented *Astragalus* administration and 5-ASA administration as the comparison. The results proved that FA was more effective in improving the symptoms of colitis. In addition, we also found an upregulated level of MPO activity in colon tissue of DSS-treated mice, which is a marker of increased inflammation and underlying permeation of immune cells. Analysis of immunoglobulin levels indicated that excessive IgE production suggests activation of mast cells and further inflammatory aggravation (48), while a reduced IgA production suggests possible loss of mucosal protection as also reported previously (49). Nevertheless, the decreased levels of MPO activity, IgE along with increased IgA could be observed after FA administration. These results were similar to Sharma et al. (16). They found that a plant-based diet could significantly reduce MPO activity and IgE content as well as increase IgA content in colitis. It is worth noting that the inhibitory effect of FA to reduce MPO activity and IgE contents appeared equivalent to 5-ASA in the current study. The balance of Th1/Th2/Th17/Treg related cytokines plays a key role in the function of the immune system. These cytokines maintain normal inflammatory homeostasis. Although this balance is strictly regulated, inflammatory mediators or damage can often lead to an imbalance of this system, which exacerbates UC (14, 50, 51). In this study, DSS-treated mice showed increased cytokine levels of Th1 (TNF-α, IL-1β, IL-6) and Th17 (IL-17). Simultaneously, decreased Th2 (IL-10) and Treg (TGF-β) cytokines levels were also observed, suggesting a Th1/Th2/Th17/Treg related cytokines imbalance in developing UC. Notably, FA significantly reversed this situation, inhibiting the production of Th1/Th17 related cytokines (TNF-α, IL-1β, IL-6 and IL-17) and increasing the production of IL-10 and TGF-β. Previously, Zhang et al. (2) and Li et al. (52) observed similar effects of fermented foxtail millet and gallic acid treatment on the DSS-induced UC. These findings suggest that regulation of the inflammatory status through cytokines balance may be one of the reasons for the apparent effect of FA in preventing UC.

The gut microbiota plays an important role in the host’s intestinal immune system. In short, the gut microbiota directs the immune system to function, and changes in the immune system feed back to the gut microbiota (7). As UC is an immune disease, it is not surprising that alterations in the intestinal microbiota can lead to intestinal inflammation and impairment of the intestinal mucosal barrier. Thus restoring the homeostasis of the microbiota is vital for the treatment of UC (53). Previous studies have found that the diversity of gut microbes decreases during colitis (2, 11, 19). Indeed, we found the reduced intestinal microbial diversity and richness of mice after DSS treatment, which was specifically manifested by the decrease of observed species, Chao-1, Shannon index and Simpson index. Conversely, both A and FA increased these indices compared to DSS-challenged mice, whereas FA seemed to reach the same levels as normal mice. Interestingly, we found that 3 weeks after 5-ASA administration, the observed species, Chao-1, Shannon index and Simpson index all decreased, indicating that the richness and diversity of microbiota were reduced. This result is consistent with the results of Wu et al. (54) and Chen et al. (55), wherein 5-ASA can reduce intestinal mucosal bacteria. This may partly explain why 5-ASA has adverse effects on the gastrointestinal tract. Therefore, the change degree of intestinal microbiota diversity may be one of the differences between A/FA and western medicine 5-ASA. On the other hand, it is well established that diet can intervene in the immune system by regulating the gut microbiota composition and metabolites such as SCFAs, indoles and bile acids (56). Not surprisingly, FA displayed excellent prebiotic properties, including inhibiting the proliferation of conditioned pathogenic bacteria and promoting the growth of beneficial bacteria. Our current work found that FA reduced the relative abundance of the pathogenic bacteria *Allobaculum*, *Shigella* and *Oscillospira*. These pathogenic bacteria were positively correlated with MPO, TNF-α, IL-1β, IL-6, IL-17, and IgE. Similar to Yuan et al.’s study (57), Isoorientin can inhibit the growth of pathogenic bacteria such as *Oscillospira* and *Shigella* which may reduce inflammation. Also, in contrast to previous studies, we found significant increases in *Bacteroides* in the gut of DSS-challenged mice. We hypothesize that this phenomenon may be caused by the proliferation of harmful species such as *Bacteroides* fragilis. It has been shown previously that cellular events triggered by *Bacteroides* fragilis toxins lead to mucosal inflammation and potential metastasis (55). On the contrary, FA significantly increased the relative abundance of *Akkermansia*, while the abundance of *Akkermansia* was quite low after unfermented *Astragalus* administration. Meanwhile, we found that *Akkermansia* was positively correlated with the production of butyric acid and isobutyric acid. Previous studies have shown that *Akkermansia* can produce acetic acid and propionic acid, and the cross-feeding of *Akkermansia* and butyric acid-producing bacteria promote butyric acid production (58). Wu et al. (11) and Li et al. (52) similarly found that *Akkermansia*, which was positively associated with butyric acid, increased in the intestinal tract of mice with colitis after Epigallocatechin-3-gallate and gallic acid supplementation. Additional studies have shown that SCFAs, represented by butyric acid, can directly affect the immune response of autoimmune CD8 + T cells, thereby regulating the downstream cytokines of T cells (59). In this study, *Akkermansia* was found to be positively correlated mainly with the anti-inflammatory cytokines IL-10 and IgA, suggesting that *Akkermansia* protected the intestinal mucosal barrier through the immune system. In addition, *Alistipes* was found as another marker microbe after FA administration, which was positively correlated with the contents of acetic acid, propionic acid, butyric acid and isovaleric acid. Although it is not clear what interactions *Alistipes* may have with immune system, our study suggests that *Alistipes* negatively correlated with the pro-inflammatory cytokines TNF-α and IL-6. Previous studies have also reported a significant reduction in disease severity when *Alistipes* was given to mice with colitis (60, 61). In summary, prophylactic FA has a unique regulatory effect on the intestinal microbiota structure and function in DSS-induced UC, and this effect may further influence intestinal mucosal inflammation and immune process.

In normal physiological conditions, the intestinal mucosa is a well-functioning barrier that separates the intestinal lumen from the internal environment of the organism. When the organism is in UC, the immune function of the mucosal barrier is abnormally activated, and the intestinal epithelium is in a state of inflammatory cell infiltration. At the same time, the gut microbiota homeostasis associated with immunity is disrupted. And pathogenic bacteria colonizing the intestine are able to act as antigens to further influence the inflammatory status. The imbalance of the inflammatory status and the gut microbiota homeostasis ultimately leads to mechanical barrier damage of the intestinal mucosa. Therefore, we investigated the mechanical barrier of intestinal mucosa after prophylactic FA administration, expecting to reflect the effect of FA on regulating the inflammatory status and microbiota. As expected, the expression of tight junction protein (ZO-1, occludin), mucous secreting protein (MUC2) genes was reduced in DSS-treated mice. This fact indicates impaired mechanical connection structures between IECs. We found that FA administration effectively reversed DSS-induced reduction in the expression levels of tight junction proteins and mucous secreting protein. Yet unfermented *Astragalus* did not seem to alter this condition. These results are similar to previous studies, wherein the lycium barbarum polysaccharide and pea seeds can alleviate colitis, as evidenced by increased tight junction protein and mucous secreting protein expression (16, 62). In addition, we detected increased DAO activity in the serum of DSS-induced mice, suggesting that damaged IECs allow the release of intracellular enzymes into the bloodstream. Similarly, increased nucleosomes level in tissues also demonstrated massive apoptosis of IECs. We further examined the expression of pre-apoptosis/anti-apoptosis genes (Bax, Bcl-2). Consistent with previous studies, the expression of the anti-apoptosis gene Bcl-2 was reduced in DSS-treated mice, while the expression of the pre-apoptosis gene Bax was increased simultaneously (13, 16). Our current study found that FA can effectively reduce apoptosis in IECs, as reflected by reduced DAO activity in serum, reduced nucleosomes in tissues and regulated apoptosis-related genes. Together, these evidences confirm that FA can indeed regulate the inflammatory status and gut microbiota to effectively repair the intestinal mucosal barrier, resulting in a strong mechanical protection.

Collectively, our results suggest that fermentation is an effective way to optimize the disease suppression ability of *Astragalus*. Compare to A, FA produced more small-molecule metabolites that were readily utilized by the organism, and these metabolites had positive meaning for FA to enhance the ability to against UC. FA intervened the intestinal inflammatory status by regulating Th1/Th2/Th17/Treg related cytokines. Meanwhile, FA-mediated enrichment of microbial community, especially *Akkermansia* and *Alistipes*, played a key role in the alleviation of colitis, which could be the major driving force behind the differential outcomes between FA and A administration. These increased populations of SCFAs-producing microbes and their metabolites induced by FA administration might also be involved in maintaining the intestinal mucosal barrier by affecting mucosal immunity. As a direct result, FA repaired the intestinal mucosal mechanical barrier and further helped protect the intestine from damage. Although the potential of FA for improving UC has been elucidated, unanswered issues about FA still exist. For example, our current study found that FA was able to attenuate the apoptosis of IECs induced by UC. However, it is not clear whether this situation is caused by FA or by FA-affected gut microbes. In addition, the way FA affects IECs apoptosis needs a comprehensive investigation in the future to fully illustrate the exact mechanism. This study may provide a scientific basis for the clinical application of FA in the prevention and therapy of UC, and facilitate UC and other inflammatory disorders.

## Data availability statement

The data presented in this study are deposited in the NCBI repository, accession number PRJNA882014, available at https://www.ncbi.nlm.nih.gov/sra/PRJNA882014.

## Ethics statement

This animal study was reviewed and approved by the Ethics Committee of Lanzhou University.

## Author contributions

JuL, PY, and CZ: research design. JuL, YW, and ZH: data analyses. JuL, YM, XL, YW, ZH, YL, HY, and JiL: research. JuL: writing the manuscript. JuL, YM, XL, YW, ZH, YL, JiL, HY, PY, and CZ: revision of results and manuscript content. ZZ, PY, and CZ: supervision. All authors contributed to the article and approved the submitted version.

## Footnotes

1
https://proteowizard.sourceforge.io/

2
http://www.gov.cn/gongbao/content/2017/content_5219148.htm
