# Supplementary material for: Fermented Astragalus and its metabolites regulate inflammatory status and gut microbiota to repair intestinal barrier damage in dextran sulfate sodium-induced ulcerative colitis
Source: Front Nutr. 2022 Nov 14;9:1035912. doi: 10.3389/fnut.2022.1035912 (PMC9702530; doi:10.3389/fnut.2022.1035912)
Supplement: Supplementary file 1 [file Data_Sheet_1.docx]

Supplementary Material

**Fermented Astragalus and its metabolites regulate inflammatory status and gut microbiota to repair intestinal barrier damage in DSS-induced ulcerative colitis**

**Supplementary Table 1:**  **16S rRNA sequencing sequence of LZU-S-ZCJ strain**

| LZU-S-ZCJ |
| --- |
| TTAGGCGCTGGTTCTAAAGGTTACCCCACCGACTTTGGGTGTTACAAACTCTCATGGTGTGACGGGCGGTGTGTACAAGGCCCGGGAACGTATTCACCGCGGCATGCTGATCCGCGATTACTAGCGATTCCGACTTCATGTAGGCGAGTTGCAGCCTACAATCCGAACTGAGAATGGCTTTAAGAGATTAGCTTACTCTCGCGAGTTCGCAACTCGTTGTACCATCCATTGTAGCACGTGTGTAGCCCAGGTCATAAGGGGCATGATGATTTGACGTCATCCCCACCTTCCTCCGGTTTGTCACCGGCAGTCTCACCAGAGTGCCCAACTTAATGCTGGCAACTGATAATAAGGGTTGCGCTCGTTGCGGGACTTAACCCAACATCTCACGACACGAGCTGACGACAACCATGCACCACCTGTATCCATGTCCCCGAAGGGAACGTCTAATCTCTTAGATTTGCATAGTATGTCAAGACCTGGTAAGGTTCTTCGCGTAGCTTCGAATTAAACCACATGCTCCACCGCTTGTGCGGGCCCCCGTCAATTCCTTTGAGTTTCAGCCTTGCGGCCGTACTCCCCAGGCGGAATGCTTAATGCGTTAGCTGCAGCACTGAAGGGCGGAAACCCTCCAACACTTAGCATTCATCGTTTACGGTATGGACTACCAGGGTATCTAATCCTGTTTGCTACCCATACTTTCGAGCCTCAGCGTCAGTTACAGACCAGACAGCCGCCTTCGCCACTGGTGTTCTTCCATATATCTACGCATTTCACCGCTACACATGGAGTTCCACTGTCCTCTTCTGCACTCAAGTTTCCCAGTTTCCGATGCACTTCTTCGGTTGAGCCGAAGGCTTTCACATCAGACTTAAAAAACCGCCTGCGCTCGCTTTACGCCCAATAAATCCGGACAACGCTTGCCACCTACGTATTACCGCGGCTGCTGGCACGTAGTTAGCCGTGGCTTTCTGGTTAAATACCGTCAATACCTGAACAGTTACTCTCAGATATGTTCTTCTTTAACAACAGAGTTTTACGAGCCGAAACCCTTCTTCACTCACGCGGCGTTGCTCCATCAGACTTTCGTCCATTGTGGAAGATTCCCTACTGCTGCCTCCCGTAGGAGTTTGGGCCGTGTCTCAGTCCCAATGTGGCCGATTACCCTCTCAGGTCGGCTACGTATCATTGCCATGGTGAGCCGTTACCTCACCATCTAGCTAATACGCCGCGGGACCATCCAAAAGTGATAGCCGAAGCCATCTTTCAAACTCGGACCATGCGGTCCAAGTTGTTATGCGGTATTAGCATCTGTTTCCAGGTGTTATCCCCCGCTTCTGGGCAGGTTTCCCACGTGTTACTCACCAGTTCGCCACTCACTCAAATGTAAATCATGATGCAAGCACCAATCAATACCAGAGTTCGTCGACTGC |

**Supplementary Table 2:**  **16S rRNA sequencing sequence of LZU-J-TSL6 strain**

| LZU-J-TSL6 |
| --- |
| ATCTGTATCTTAGGCGGCTGGTTCCTAAAAGGTTACCCCACCGACTTTGGGTGTTACAAACTCTCATGGTGTGACGGGCGGTGTGTACAAGGCCCGGGAACGTATTCACCGCGGCATGCTGATCCGCGATTACTAGCGATTCCGACTTCATGTAGGCGAGTTGCAGCCTACAATCCGAACTGAGAATGGCTTTAAGAGATTAGCTTACTCTCGCGAGTTCGCAACTCGTTGTACCATCCATTGTAGCACGTGTGTAGCCCAGGTCATAAGGGGCATGATGATTTGACGTCATCCCCACCTTCCTCCGGTTTGTCACCGGCAGTCTCACCAGAGTGCCCAACTTAATGCTGGCAACTGATAATAAGGGTTGCGCTCGTTGCGGGACTTAACCCAACATCTCACGACACGAGCTGACGACAACCATGCACCACCTGTATCCATGTCCCCGAAGGGAACGTCTAATCTCTTAGATTTGCATAGTATGTCAAGACCTGGTAAGGTTCTTCGCGTAGCTTCGAATTAAACCACATGCTCCACCGCTTGTGCGGGCCCCCGTCAATTCCTTTGAGTTTCAGCCTTGCGGCCGTACTCCCCAGGCGGAATGCTTAATGCGTTAGCTGCAGCACTGAAGGGCGGAAACCCTCCAACACTTAGCATTCATCGTTTACGGTATGGACTACCAGGGTATCTAATCCTGTTTGCTACCCATACTTTCGAGCCTCAGCGTCAGTTACAGACCAGACAGCCGCCTTCGCCACTGGTGTTCTTCCATATATCTACGCATTTCACCGCTACACATGGAGTTCCACTGTCCTCTTCTGCACTCAAGTTTCCCAGTTTCCGATGCACTTCTTCGGTTGAGCCGAAGGCTTTCACATCAGACTTAAAAAACCGCCTGCGCTCGCTTTACGCCCAATAAATCCGGACAACGCTTGCCACCTACGTATTACCGCGGCTGCTGGCACGTAGTTAGCCGTGGCTTTCTGGTTA |

**Supplementary Table 3: Primers for detection of relative abundance of genes related to** **colonic mucous barrier integrity, proliferation and apoptosis of** **intestinal epithelial cells**

| **Gene Name** | **Forward Primer** | **Reverse Primer** |
| --- | --- | --- |
| ZO-1 | GGGAAAACCCGAAACTGATG | GCTGTACTGTGAGGGCAACG |
| occludin | ATAATGGGAGTGAACCCGACG | CGATCCATCTTTCTTCGGGTTT |
| MUC2 | GGCTCGGAACTCCAGAAAGAAG | CTCGGCAGTCAGACGCAAAG |
| Bax | GCCTTTTTGCTACAGGGTTTCAT | TATTGCTGTCCAGTTCATCTCCA |
| Bcl-2 | GCTACCGTCGTGACTTCGCA | CATCCCAGCCTCCGTTATCC |
| GAPDH | CCTCGTCCCGTAGACAAAATG | TGAGGTCAATGAAGGGGTCGT |

**Supplementary Table 4: The normalized contents of selected metabolites associated with increased metabolites**

| **Name** | **A (Mean ± SD)** | | **FA (Mean ± SD)** | **Fold Change** | **VIP** |
| --- | --- | --- | --- | --- | --- |
| Melibiose | 217753379.72 ± 22.08 | | 66205461.40 ± 47.13 | 3.29 | 1.01 |
| Trehalose | 5161428449.54 ± 84.16 | | 26667919.80 ± 70.69 | 193.54 | 1.15 |
| Gentianose | 47583703.03 ± 39.68 | | 2566429.42 ± 32.78 | 18.54 | 1.15 |
| Stachyose | 8198681.17 ± 22.96 | | 949655.63 ± 17.86 | 8.63 | 1.06 |
| Hesperetin 7-neohesperidoside | 15321071.17 ± 18.92 | | 1609949.29 ± 58.33 | 9.52 | 1.12 |
| Peonidin-3-glucoside | 1128562613.16 ± 16.53 | | 141787519.31 ± 91.18 | 7.96 | 1.00 |
| Cyanidin 3-galactoside | 225416261.46 ± 44.50 | | 1561082.64 ± 45.71 | 144.40 | 1.15 |
| Cyanidin 3-glucoside | 50014500.12 ± 9.33 | | 486867.78 ± 52.94 | 102.73 | 1.18 |
| Gluconolactone | 35789027.22 ± 30.74 | | 5744002.36 ± 19.46 | 6.23 | 1.17 |
| D-Galactose | 404068797.43 ± 22.12 | | 5304228.75 ± 54.36 | 76.18 | 1.17 |
| Sucrose | 363642253.25 ± 13.96 | | 28546391.05 ± 75.40 | 12.74 | 1.09 |
| 3,4-Dihydroxyphenylacetaldehyde | 30625467.38 ± 12.02 | | 328457741.95 ± 29.96 | 0.09 | 1.15 |
| 3,4-Dihydroxybenzeneacetic acid | 192938024.91 ± 20.34 | | 47719208.01 ± 74.38 | 4.04 | 1.03 |
| Pipecolic acid | 277669250.49 ± 12.78 | | 98743822.25 ± 25.96 | 2.81 | 1.11 |
| 4-Aminocatechol | 1087163729 ± 6.49 | | 182139085.71 ± 24.42 | 5.97 | 1.16 |
| L-Glutamine | 835769170.34 ± 31.87 | | 190170828.09 ± 16.97 | 4.39 | 1.10 |
| Dodecanedioic acid | 63047825.25 ± 4.24 | | 18515704.07 ± 26.34 | 3.41 | 1.13 |
| Deoxyuridine | 38636951.61 ± 11.82 | | 2220601.06 ± 15.84 | 17.41 | 1.17 |
| 2-Keto-6-aminocaproate | 1089006319.79 ± 4.86 | | 12077142.38 ± 122.74 | 90.17 | 1.08 |
| Guanidoacetic acid | 12939742.66 ± 41.64 | | 2897397.88 ± 16.26 | 4.47 | 1.10 |
| 21-Hydroxypregnenolone | 67872918.69 ± 22.99 | | 28526636.40 ± 17.31 | 2.38 | 1.10 |
| 17a-Hydroxypregnenolone | 581363571.76 ± 22.29 | | 23294475.04 ± 32.51 | 24.96 | 1.16 |
| Uracil | 13994402.79 ± 69.63 | | 1221937.42 ± 21.88 | 11.45 | 1.08 |
| Uridine 2',3'-cyclic phosphat | 232427922.50 ± 18.08 | | 27386992.34 ± 29.49 | 8.49 | 1.15 |
| UMP | | 16000337.68 ± 10.16 | 248970.70 ± 64.07 | 64.27 | 1.16 |
| Thymine | | 65417428.91 ± 15.54 | 30911855.57 ± 31.03 | 2.12 | 1.01 |
| L-Lysine | | 760228449.73 ± 29.55 | 267216550.51 ± 27.66 | 2.84 | 1.08 |


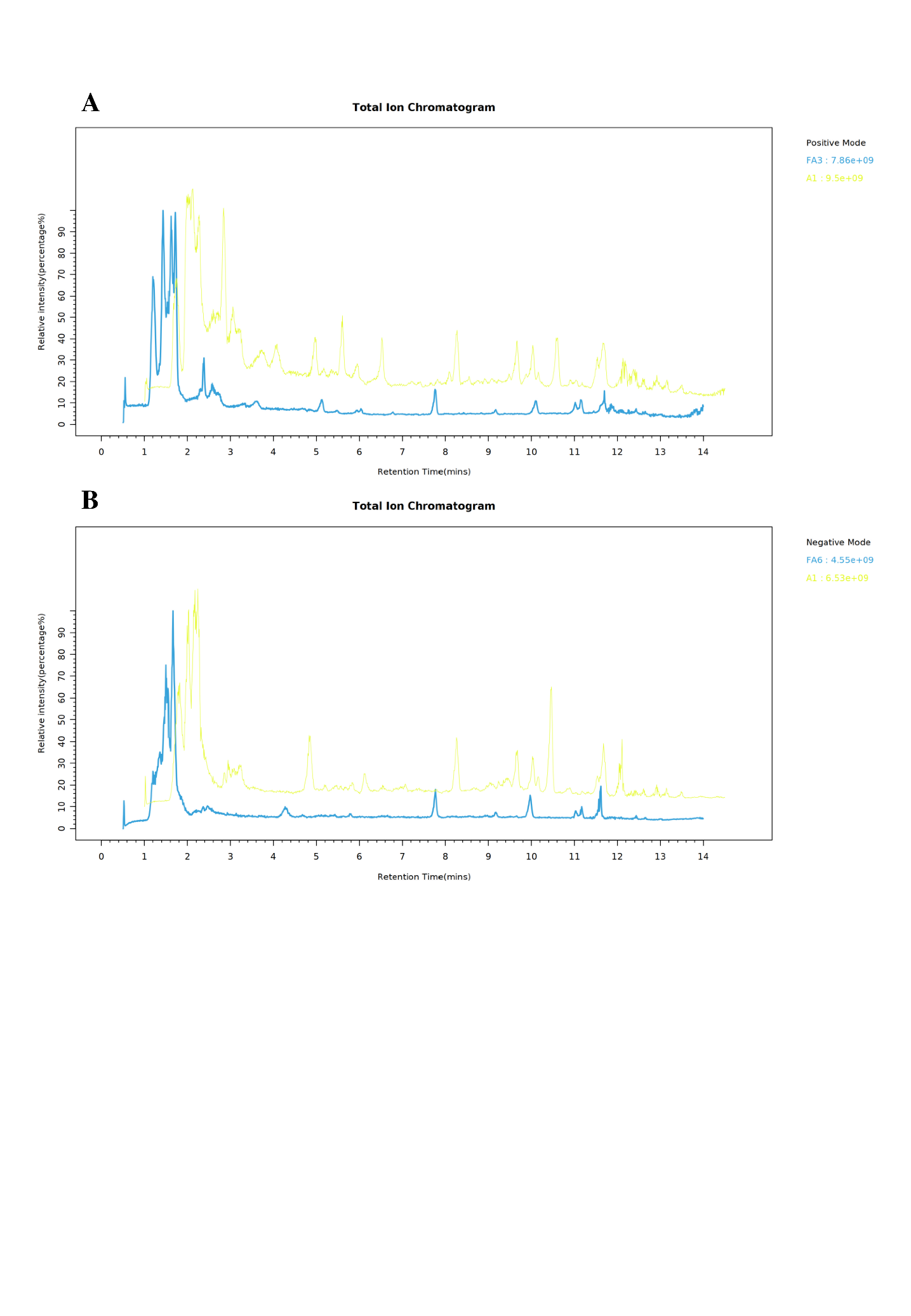


**Supplementary Figure 1:** **The total ion chromatogram of A and FA: (A)** positive ion mode; **(B)** negative ion mode.


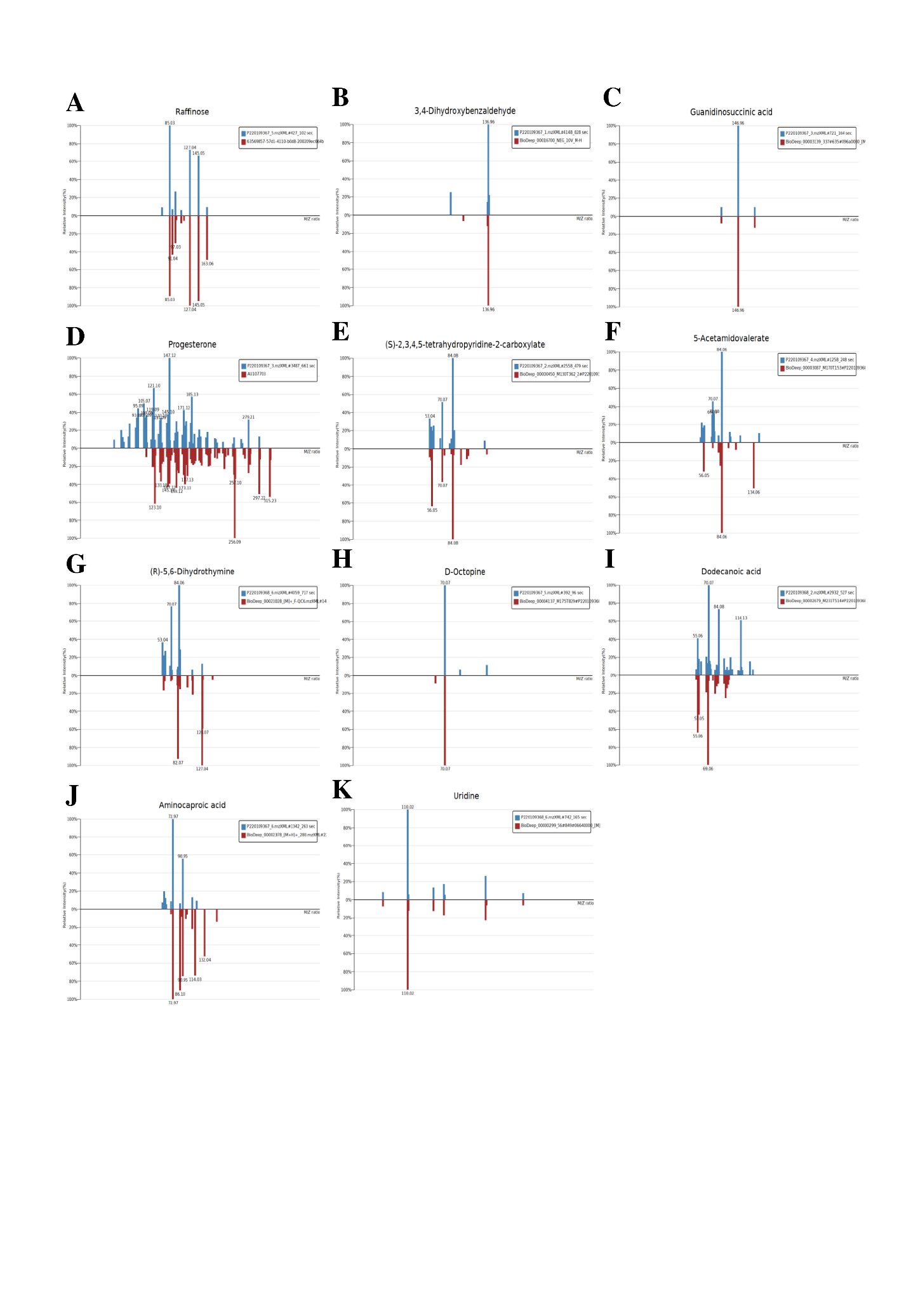


**Supplementary Figure 2:** **The mass identification spectrograms of 11 differential metabolites: (A)** Raffinose; **(B)** 3,4-Dihydroxybenzaldehyde; **(C)** Guanidinosuccinic acid; **(D)** Progesterone; **(E)** (S)-2,3,4,5-tetrahydropyridine-2-carboxylate; **(F)** 5-Acetamidovalerate; **(G)** (R)-5,6-Dihydrothymine; **(H)** D-Octpine; **(I)** Dodecanoic acid; **(J)** Aminocaproic acid; **(K)** Uridine.
